# Association between dental fear and oral health habits and treatment need among University students in Finland: a national study

**DOI:** 10.1186/s12903-016-0179-y

**Published:** 2016-02-27

**Authors:** Vesa Pohjola, Aino Rekola, Kristina Kunttu, Jorma I. Virtanen

**Affiliations:** Research Unit of Oral Health Sciences, Faculty of Medicine, University of Oulu, Oulu, Finland; Medical Research Center Oulu, Oulu University Hospital, Oulu, Finland; Finnish Student Health Service, Turku, Finland

**Keywords:** Dental fear, University students, Dental health, Tooth brushing, Attitude to food

## Abstract

**Background:**

First-year university students are in a new, independent life situation, which may affect health behaviour, including oral health habits. The aim of this study was to evaluate the association between dental fear and oral health habits, while considering the simultaneous effects of attitude toward food and treatment need at dental check-ups.

**Methods:**

The data (*n* = 8514) for this national cross-sectional study were collected from health registers of Finnish Student Health Service. As part of health examination all first-year university students in Finland were sent an electronic questionnaire asking about general, psychological and oral health, and health habits. Dental fear was measured by the question: “How afraid are you of visiting a dentist?” (reply alternatives: “Not at all”, “Somewhat” and “Very”). Chi-square tests and Multiple logistic regression analyses were used to determine the associations between dental fear and oral health habits (tooth brushing, tobacco use, frequency of eating and drinking, eating habits and interval between dental check-ups) as well as attitude to food and treatment need at dental check-ups while controlling for age, gender, general mood and feelings in social situations.

**Results:**

Of the oral health habits, tooth brushing and tobacco use were associated with dental fear. Those who brushed their teeth once a day or less often or used tobacco regularly were more likely to have high dental fear than those who brushed their teeth twice a day or more often or used tobacco occasionally or not at all. Students who reported not having a normal attitude to food were more likely to have high dental fear than were those reporting normal attitude to food, but the frequency of eating and drinking was not associated with dental fear. Students who reported needing treatment frequently or at every dental check-up were more likely to have high dental fear than those who reported rarely or never needing treatment.

**Conclusion:**

Those students with high dental fear seem to be at risk of having poor oral health habits and abnormal attitude to food, which may increase the risk of deterioration of oral health and the need for treatment. Dental teams should make efforts in helping fearful patients to find motivation for good oral health habits.

## Background

First-year university students are in transition to adulthood and this new, independent life situation may affect their health behaviour [1–4] i.e., eating habits, smoking and regularity of dental care.

An association between dental fear and dental attendance has been found in many previous studies; those with high dental fear are more likely to visit a dentist irregularly than are those with lower level of dental fear [5–7]. People with dental fear also tend to visit a dentist on a problem-oriented basis rather than for regular check-ups [8]. Treatment need is more often found among those with high dental fear than among those with no or low level of dental fear [5, 9–12].

The association between dental fear and dental self-care is not as clear as the association between dental fear and dental attendance. Some studies have shown that the level of oral hygiene is poorer among people with high dental fear than among those with low dental fear [13, 14], but there are also opposite findings [5]. Among adults, those who brushed their teeth less than twice a day were more likely to have high dental fear than were those who brushed at least twice a day; however, this was not observed in all age groups [10].

Oral hygiene and smoking have great impact on oral health [15, 16]. In some previous studies dental fear has been related to smoking, those who smoke regularly being more likely to report high dental fear than those who smoke occasionally or not at all [12, 17]. Association between dental fear and tobacco use and alcohol use was observed in a recent study among Finnish university students [18]. Among adults with dental fear, smoking and tooth brushing have been found to predict dental attendance [19].

In addition to dental attendance, dental self-care and smoking, eating habits and attitude to food can also affect oral health. Oral health and dental fear are known to be associated [5, 9–12], but the possible associations between dental fear and eating habits and/or food attitude remain obscure. Trends in eating habits, e.g., frequent snacking, consumption of sugar-sweetened beverages, and desire to be slim [20, 21] may affect general- and dental health [20, 22]. There is also some evidence that people with eating disorders have higher levels of dental fear than do the general population [23, 24].

Several other psychological problems (e.g., anxiety and depressive disorders) are associated with dental fear [25, 26] as well as with dental attendance [27], cigarette smoking [28], and eating habits [29]. In terms of dental fear differences are known to exist between gender, age - and socio-economic groups [30, 31], dental attendance [32], dental self-care [33], smoking [34, 35] and eating habits [36, 37]. Age, gender, socio-economic status and psychological problems should be taken into account when the association between dental fear and oral health-related behaviour is studied.

The aim of this study was to evaluate simultaneously the association between dental fear and oral health habits (tooth brushing, frequency of eating and drinking, eating habits, tobacco use and interval between dental check-ups), attitude toward food and treatment need at dental check-up, when controlling for age, gender, general mood and feelings in social situations. The study was conducted among first-year university students in Finland. The study hypothesis was that high dental fear is more common among those with poor oral health-related habits, abnormal attitude to food, long interval between dental check-ups, and regular treatment need at check-ups compared to those with good oral health-related habits, normal attitude to food, short interval between check-ups, and low level of treatment need at check-ups.

## Methods

Finnish university students are entitled to subsidized health care services, including dental care, provided by Finnish Student Health Service (FSHS). The data for this cross-sectional study were collected anonymously form health registers of the FSHS. As a part of their health examination all first-year university students were sent an electronic health questionnaire inquiring about their general, psychological and oral health, and health habits. If the inquiry showed problems in the student’s health or health habits, he/she was invited to a health examination. Each student received a personal reply to the electronic health questionnaire. The questionnaire was e-mailed in Finnish, Swedish or English. Finnish and Swedish are official languages in Finland; however, less than 4 % of the university students in Finland are international students. The data for this study were collected from electronic health inquiries answered by university students who started their studies in autumn 2011 and responded to the health questionnaire by the end of February 2012. All universities in Finland were included in the study. In 2011 there were 17 091 first-year students in Finland. The electronic health inquiry was answered by 8514 students, i.e., 50 % of the first-year students; 34.7 % of those who answered were men (Table 1). The prevalence of high dental fear was 5.4 % [18]. Permission for the study was given by the health register authorities of the FSHS taking care that the information in the registers is used according to medical laws and ethics. The study was approved by the Ethics Committee of the Northern Ostrobothnia Hospital District (EETTMK 101/2013 § 238).

**Table 1:** Description of the participants (*n* = 8514) according to background factors^a^

	*n*	All %	Men %	Women %
Gender				
Men	2958	34.7		
Women	5556	65.3		
Age				
≤ 19	2516	29.6	19.9	34.7
20	2150	25.3	30.3	22.6
21–24	2164	25.4	30.1	22.9
25–29	927	10.9	12.4	10.1
30–39	481	5.6	5.2	5.9
40+	276	3.2	2.1	3.9
General mood				
Positive (1 to +10)	563	89.7	89.6	89.8
Neutral (0)	310	3.6	4.3	3.3
Negative (−10 to −1)	7641	6.6	6.2	6.8
Feelings in social situations				
Positive (1 to +10)	6133	72.0	74.2	70.9
Neutral (0)	617	7.2	7.9	6.9
Negative (−10 to −1)	1764	20.7	17.9	22.2

^a^Gender, age, general mood and feelings in social situations

In the electronic inquiry, dental fear was covered by a single question: “How afraid are you of visiting a dentist?”. The alternatives for replying were “Not at all”, “Somewhat” and “Very”. This question of dental fear has been validated among Norwegian and Finnish adult populations [38, 39]. The interval between dental check-ups was determined by the question: “When was your last visit to a dentist for a check-up?”. The reply alternatives were “0–2 years ago”, “3–5 years ago” and “over 5 years ago”. Treatment need was determined with the question: “At dental check-ups, do you usually have cavities that require fillings”, with three reply alternatives: “Never”, “Rarely” and “Frequently or every time”. The frequency of tooth brushing was ascertained using the question: “How many times a day do you usually brush your teeth?”. The alternatives for replying were “Twice a day or more often”, “Once a day” and “Less than once a day”. The regularity of smoking or using snuff was determined using the question: “Do you smoke or use snuff?”. The alternatives for replying were “Regularly”, “Occasionally” and “Not at all”. The questions about dental fear and dental check-up have been used e.g., in the Health 2000 study in Finland [40] and the questions about frequency of tooth brushing and tobacco use have been utilized e.g., in the Finnish Student Health Survey 2012 [41].

Frequency of eating and drinking during a day was determined by the question: “How many times a day do you usually eat or drink (drinking water, coffee or tea without sugar does not count)?”. The alternatives for replying were “Six times a day or less”, “Seven to ten times a day” and “More than ten times a day”. Healthy eating habits were determined using the question: “How healthy would you evaluate your eating habits?”. The reply alternatives were on the scale −10 to +10, where answers 1 to +10 indicated “Healthy eating habits”, 0 “Do not know” and −10 to −1 “Unhealthy eating habits”. Attitude to food was determined using the question: “Do you have a normal attitude to food?”, with three reply alternatives: “Yes”, “Do not know” and “No”. The questions about frequency of eating and drinking and attitude to food have been used in previous studies in FSHS [41].

General mood was ascertained with the question: “How is your general mood?”. The reply alternatives were on the scale −10 to +10, where answers 1 to +10 indicated “Positive mood”, 0 “Neutral mood” and answers −10 to −1 “Negative mood”. Feelings in social situations was determined using the question: “How do you feel in social situations (e.g., giving an oral presentation)?”. The alternatives for replying were on the scale −10 to +10 and answers 1 to +10 indicated “Positive feelings”, 0 “Neutral feelings” and −10 to −1 “Negative feelings”. The questions about feelings in social situations and general mood have been used in many studies in FSHS since 1979 [41].

For the analyses, age was categorized into six groups: 19 or younger, 20, 21–24, 25–29, 30–39 and 40+. The youngest age group had entered the university directly from high school, the second age group entered one year after high school (boys often after military service) and the other age groups may have been working or studying something else before entering the university.

### Statistical analysis

Bivariate association between dental fear and age, gender, tooth brushing, regularity of smoking or using snuff, frequency of eating or drinking a day, healthy eating habits, attitude to food, interval between dental check-ups and treatment need at check-up were evaluated. The statistical significances of differences in the bivariate associations were evaluated with Chi-square tests. Multiple logistic regression analyses were used to evaluate the association between dental fear and tooth brushing, smoking or using snuff, frequency of eating and drinking, healthy eating habits and attitude to food, interval between dental check-ups and treatment need at check-up, controlling for age, gender, general mood and feelings in social situations. Age was entered into the multiple logistic regression analysis as a continuous variable. The level of statistical significance was set at *p* < 0.05. The analysis was performed with SPSS, version 20.0 [42].

## Results

Twice a day tooth brushing was less frequent among men than among women (Table 2). Men more commonly than women reported regular smoking or using snuff. Men more often than women also reported unhealthy eating habits and eating or drinking more than ten times a day. Among women abnormal attitude to food was more common than among men. Men more commonly than women reported having last visited a dentist for a check-up five years or more ago, but women more frequently reported needing treatment at dental check-ups.

**Table 2:** Description of the participants (*n* = 8514) according to oral health-related factors^a^

Dental fear	*n*	All %	Men %	Women %
Very afraid	459	5.4	2.5	6.9
Somewhat afraid	2836	33.3	25.0	37.7
Not at all afraid	5219	61.3	72.5	55.3
Tooth brushing				
Twice a day or more often	6190	72.7	58.7	80.2
Once a day	2203	25.9	38.4	19.2
Less than once a day	121	1.4	2.9	0.6
Smoking or using snuff				
Not at all	6700	78.7	72.9	81.8
Occasionally	1275	15.0	17.6	13.6
Regularly	539	6.3	9.5	4.6
Eating or drinking (something other than water, coffee/tea without sugar)				
> 10 times a day	124	1.5	2.5	0.9
7–10 times a day	1572	18.5	23.6	15.7
≤ 6 times a day	6818	80.1	73.9	83.4
Eating habits				
Healthy (1 to +10)	7246	85.1	82.3	86.6
Do not know (0)	420	4.9	6.0	4.4
Not healthy (−10 to −1)	848	10.0	11.7	9.0
Attitude to food				
Abnormal	308	3.6	1.1	4.9
Do not know	964	11.3	5.9	14.2
Normal	7242	85.1	92.9	80.9
Last check-up				
> 5 years ago	460	5.4	7.3	4.4
3–5 years ago	2472	29.0	28.9	29.1
0–2 years ago	5582	65.6	63.8	66.5
Treatment need at dental check-ups				
Frequently or every time	1990	23.4	19.6	25.4
Rarely	4261	50.0	52.1	49.0
Never	2263	26.6	28.3	25.7

^a^Dental fear, last check-up, treatment need at dental check-up, tooth brushing, eating and drinking frequency, eating habits, attitude to food and tobacco use

The prevalence of high dental fear was higher among students who reported brushing their teeth ones a day or less often than among those who reported brushing their teeth at least twice a day (Table 3). High dental fear was also more common among students who had unhealthy eating habits than among those who had healthy eating habits. The prevalence of high dental fear was similar among students who reported eating or drinking more than ten times a day compared to those who reported eating or drinking ten times a day or less. Students who reported that their attitude to food was abnormal more often had high dental fear than did those who reported normal attitude to food. Regular smokers and users of snuff more often reported high dental fear than did occasional or non-smokers or users of snuff. The prevalence of high dental fear was higher among students who had last visited a dentist for a check-up more than 5 years ago than among those who had been to a dental check-up less than 5 years ago. High level of dental fear was more common among students who frequently or every time had treatment need at dental check-ups than among those students who never or rarely needed treatment.

**Table 3:** Gender-specific prevalence of dental fear among first-year university students in Finland according to oral health-related factors^a^

	Dental fear among all				Dental fear among women				Dental fear among men			
	Very afraid	Somewhat afraid	Not at all afraid	*p* ^b^	Very afraid	Somewhat afraid	Not at all afraid	*p* ^b^	Very afraid	Somewhat afraid	Not at all afraid	*p* ^b^
	% (*n*)	% (*n*)	% (*n*)		% (*n*)	% (*n*)	% (*n*)		% (*n*)	% (*n*)	% (*n*)	
Tooth brushing												
Twice a day or more often	5.2 (324)	34.0 (2104)	60.8 (3762)	0.095	6.5 (288)	37.7 (1677)	55.9 (2489)	0.078	2.1 (36)	24.6 (427)	73.3 (1273)	0.001
Once a day	5.6 (124)	31.5 (695)	62.8 (1384)		8.8 (94)	38.1 (407)	53.0 (566)		2.6 (30)	25.4 (288)	72.0 (818)	
Less than once a day	9.1 (11)	30.6 (37)	60.3 (73)		8.6 (3)	34.3 (12)	57.1 (20)		9.3 (8)	29.1 (25)	61.6 (53)	
Do you smoke or use snuff?												
Not at all	5.0 (334)	33.3 (2231)	61.7 (4135)	<0.001	6.4 (289)	37.2 (1692)	56.4 (2564)	<0.001	2.1 (45)	25.0 (539)	72.9 (1571)	0.002
Occasionally	5.8 (74)	33.3 (425)	60.9 (776)		8.1 (61)	40.3 (304)	51.6 (389)		2.5 (13)	23.2 (121)	74.3 (387)	
Regularly	9.5 (51)	33.4 (180)	57.1 (308)		13.6 (35)	38.9 (100)	47.5 (122)		5.7 (16)	28.4 (80)	66.0 (186)	
Eating or drinking (something other than water, coffee or tea without sugar)												
≤6 times a day	5.5 (374)	33.3 (2272)	61.2 (4172)	0.956	6.8 (315)	37.5 (1736)	55.7 (2582)	0.448	2.7 (59)	24.5 (536)	72.8 (1590)	0.177
7–10 times a day	5.0 (79)	33.2 (522)	61.8 (971)		7.4 (65)	39.5 (345)	53.1 (464)		2.0 (14)	25.4 (177)	72.6 (507)	
>10 times a day	4.8 (6)	33.9 (42)	61.3 (76)		10.2 (5)	30.6 (15)	59.2 (29)		1.3 (1)	36.0 (27)	62.7 (47)	
Eating habits (−10 to +10)												
Healthy (+1 to +10)	5.0 (365)	33.0 (2393)	61.9 (4488)	0.001	6.5 (311)	37.3 (1795)	56.2 (2705)	0.001	2.2 (54)	24.6 (598)	73.2 (1783)	0.019
Do not know (0)	5.7 (24)	36.0 (151)	58.3 (245)		8.6 (21)	42.8 (104)	48.6 (118)		1.7 (3)	26.6 (47)	71.8 (127)	
Not healthy (−10 to −1)	8.3 (70)	34.4 (292)	57.3 (486)		10.6 (53)	39.2 (197)	50.2 (252)		4.9 (17)	27.5 (95)	67.6 (234)	
Attitude to food												
Normal	4.7 (341)	32.2 (2333)	63.1 (4568)	<0.001	6.2 (279)	37.0 (1665)	56.7 (2550)	<0.001	2.3 (62)	24.3 (668)	73.4 (2018)	<0.001
Do not know	8.4 (81)	40.1 (387)	51.5 (496)		9.1 (72)	41.1 (324)	49.7 (392)		5.1 (9)	35.8 (63)	59.1 (104)	
Abnormal	12.0 (37)	37.7 (166)	50.3 (155)		12.4 (34)	39.1 (107)	48.5 (133)		8.8 (3)	26.5 (9)	64.7 (22)	
Last dental check-up												
0–2 years ago	5.2 (288)	32.4 (1811)	62.4 (3483)	0.003	6.6 (245)	36.4 (1344)	57.0 (2106)	0.001	2.3 (43)	24.7 (467)	73.0 (1377)	0.012
3–5 years ago	5.4 (133)	34.7 (857)	60.0 (1482)		7.1 (114)	40.1 (648)	52.8 (854)		2.2 (19)	24.4 (209)	73.4 (628)	
>5 years ago	8.3 (38)	36.5 (168)	55.2 (254)		10.6 (26)	42.4 (104)	46.9 (115)		5.6(12)	29.8 (64)	64.7 (139)	
Treatment need at dental check-ups												
Never	1.9 (43)	22.1 (499)	76.0 (1721)	<0.001	2.5 (36)	25.4 (362)	72.1 (1029)	<0.001	0.8 (7)	16.4 (137)	82.8 (692)	<0.001
Rarely	4.3 (183)	33.8 (1439)	61.9 (2639)		5.8 (159)	37.9 (1030)	56.3 (1531)		1.6 (24)	26.5 (409)	71.9 (1108)	
Frequently or every time	11.7 (233)	45.1 (898)	43.2 (859)		13.5 (190)	50.0 (704)	36.6 (515)		7.4 (43)	33.4 (194)	59.2 (344)	

^a^Time to last check-up, treatment need at last check-up, tooth brushing frequency, eating or drinking frequency, eating habits, attitude to food, and smoking or using snuff

^b^ *P*-values for chi-square tests

In the logistic regression analyses (Table 4), when age, gender, general mood and feelings in social situations were controlled for, those who brushed their teeth once a day or less often were more likely to have high dental fear than were those who brushed their teeth twice a day or more often. Tobacco use was also associated with dental fear. Those who smoked or used snuff regularly were also more likely to have high dental fear than those who smoked or used snuff occasionally or not at all. Attitude toward food was associated with dental fear, but frequency of eating and drinking and healthy eating habits were not. Those reporting abnormal attitude to food were more likely to have high dental fear than were those who reported normal attitude to food. Students who reported frequent need for treatment or treatment need at every dental check-up were more likely to have high dental fear than students who reported having never or rarely treatment need at dental check-ups. When the limit for intervals between dental check-ups was set at 2 years or less, the interval between dental check-ups was not associated with dental fear. In corresponding gender-specific models among women, similar associations were found between dental fear and tooth brushing, smoking or using snuff, attitude to food and to treatment need at check-up (Table 5). Among men, similar associations were found between dental fear and smoking or using snuff and treatment need.

**Table 4:** Results of logistic regression analyses among university students in Finland (*n* = 8514)^a,b^

	*P*	OR	95 % CI	
			Lower	Upper
Treatment need at check-up^c^	<0.001	3.4	2.8	4.3
Gender^d^	<0.001	3.0	2.3	4.0
Smoking or using snuff^e^	<0.001	2.1	1.5	3.0
Attitude to food^f^	0.007	1.7	1.2	2.5
Tooth brushing^g^	0.006	1.4	1.1	1.8

Nagelkerke R2 = 0.104

*P* *P*-value, *OR* odds ratio, *CI* confidence interval

^a^Dental fear was dependent variable (0 = somewhat or not at all afraid, 1 = very afraid)

^b^Controlling for age, general mood and feelings in social situation

^c^Treatment need at check-up: 0 = rarely or never, 1 = frequently or every time

^d^Gender: 0 = male, 1 = female

^e^Smoking or using snuff: 0 = not at all, 1 = occasionally or regularly

^f^Attitude to food: 0 = Normal, 1 = Abnormal (those answering: “Do not know” were excluded)

^g^Tooth brushing: 0 = two times a day or more often, 1 = less than two times a day

**Table 5:** Gender-specific results of logistic regression analyses among university students in Finland (*n* = 8514)^a,b^

	*P*	OR	95 % CI	
			Lower	Upper
Women				
Treatment need at check-up^c^	<0.001	3.2	2.5	4.0
Smoking or using snuff^d^	0.005	1.9	1.2	2.9
Attitude to food^e^	0.014	1.7	1.1	2.5
Tooth brushing^f^	0.014	1.4	1.1	1.8
Men				
Treatment need at check-up^c^	<0.001	5.5	3.4	9.0
Smoking or using snuff^d^	0.010	2.1	1.2	3.8

*Woman* Nagelkerke R2 = 0.072

*Men* Nagelkerke R2 = 0.104

*P* *P*-value, *OR* odds ratio, *CI* confidence interval

^a^Dental fear was dependent variable (0 = somewhat or not at all afraid, 1 = very afraid)

^b^Controlling for age, general mood and feelings in social situation

^c^Treatment need at check-up: 0 = rarely or never, 1 = frequently or every time

^d^Smoking or using snuff: 0 = not at all, 1 = occasionally or regularly

^e^Attitude to food: 0 = Normal, 1 = Abnormal (those answering: “Do not know” were excluded)

^f^Tooth brushing: 0 = two times a day or more often, 1 = less than two times a day

## Discussion

Our aim in this study was to assess the association between dental fear and oral health-related habits and treatment need at dental check-up among first-year university students in Finland. Of the oral health-related habits, tooth brushing and tobacco use were associated with dental fear; those who brushed their teeth once a day or less often or used tobacco regularly were more likely to have high dental fear than those who brushed their teeth twice a day or more often or used tobacco occasionally or not at all. The attitude to food was associated with dental fear, but the frequency of eating and drinking was not. Students reporting an abnormal attitude to food were more likely to have high dental fear than were students who reported normal attitude to food. Students who reported a need for treatment frequently or at every dental check-up were more likely to have high dental fear than students who rarely or never reported treatment need at dental check-ups.

First-year university students are in transition to adulthood. This new life situation may affect their health behaviour [1–4], e.g., oral health habits. In our study, of these habits, tooth brushing was associated with dental fear. Those who brushed their teeth less than twice a day were more likely to have high dental fear than were those who brushed their teeth twice a day or more often. A similar association has previously been found e.g., among a group of undergraduate students in United States [14] and among age-group 65+ year-olds in Finland [10]. The findings of previous research are not all in agreement; e.g., among Norwegian adult population [5] and among age-group 30–64 year-olds in Finland [10] tooth brushing was not associated with dental fear.

Of the oral health habits included in this study, tobacco use was associated with dental fear. Students who smoked or used snuff regularly were more likely to have high dental fear than students who did not smoke or use snuff or who smoked or used snuff occasionally. Similar association between dental fear and tobacco use among this student population has previously been seen when alcohol use has been included in the analyses [18]. These results are also similar to those of previous studies among the general population [17, 19] and among adult patients at a university clinic [12]. Smoking habits and nicotine dependence have been associated with anxiety in general [43], and smoking after a stressful stimulus has been associated with lowered levels of anxiety [44]. Patients with high dental fear might find temporary relief for their anxiety by smoking. However, the use of tobacco predisposes people to develop anxiety over time by, e.g., producing chronic withdrawal symptoms and possible precipitation of somatic or emotional symptoms that maintain anxiety [45].

In this present study we found associations between dental fear and treatment need. Students who reported frequently or at every at dental check-up having cavities that require fillings were, e.g., among men 5.5 times, more likely to report high dental fear than were students who reported rarely or never needing treatment at check-up. This result is in agreement with previous studies. Those with high dental fear more often reported treatment need and were found to have poorer dental health than those with lower level of dental fear [5, 9–12]. In addition, the problem-oriented pattern of visiting a dentist among those with high dental fear [8] can increase the need for treatment [46]. However, in this study the interval between dental check-ups was not associated with dental fear. This finding is surprising as interval between dental check-ups may be seen as an indicator of regularity of dental care and in many earlier studies those with high dental fear have been likely to visit a dentist irregularly [5–7]. However, in a previous study among 30–34 year-olds in Finland dental attendance was also not associated with dental fear [6]. The authors speculated that in Finland, where dental care is regular and free of charge until the age of 19, people with dental fear could have learned to visit a dentist regularly in spite of their fear. If this effect of “latent inhibition” could be seen among the age-group 30–34, it should also be seen among the participants of this study. Furthermore, the results of the present study showed that the interval between dental check-ups was not associated with dental fear, but treatment need at dental check-up was. This may indicate that people with dental fear might have difficulties successfully completing treatment planned during check-up.

Eating habits can affect health, e.g., intake of sugar-sweetened beverages is a significant contributor to both general and dental health problems [20, 22]. In this study, bivariate associations were observed between eating habits and dental fear, with the prevalence of high dental fear being higher among those reporting unhealthy eating habits than among those reporting healthy eating habits. In the multivariate analyses, however, this association was no longer significant. In the bivariate analyses, the frequency of eating and drinking was similar among participants with different level of dental fear. Thus, it was not surprising that in the multivariate analyses frequency of eating and drinking was not associated with dental fear. Other factors such as the cost of food [3, 36, 47], the desire to be slim [21], frequent snacking and other trends in eating [48] might affect eating habits, frequency of eating and drinking as well as dental health. In this study dental fear and frequency of eating and drinking were not associated; thus the relationship between dental health and dental fear apparently is not affected by frequency of eating and drinking.

Eating habits and frequency of eating and drinking were not associated with dental fear in the multivariate analyses, but attitude to food was. The question concerning attitude to food used in this study has previously been used in Finland for studying eating disorders [41]. This question may measure different, possibly psychological, aspects of eating than do questions about eating habits and frequency of eating and drinking. In addition, the results of previous studies have shown that people with eating disorders have higher levels of dental fear than do the general population [23, 24]. These results indicate the importance of psychological aspects in eating and in dental fear.

In this study there are both strengths and limitations. The large national sample was collected from all universities in Finland. The electronic health inquiry was answered by half of these university students, which can be considered good participation. According to a meta-analysis, the mean response rate in the web surveys was 34 % [49]. In our study 34.7 % of the participants were men, while 44.6 % of the first year university students were men in Finland in 2011 [50]. Survey response and non-response studies have shown that women are more likely than men to participate in studies [51]. Since the population of this study included only university students, this study cannot provide information about dental fear among the rest of the young adult population in Finland. The level of dental fear was measured with a single question that has been shown to be valid and reliable in Finnish and Norwegian adult populations [38, 39]. Length of a questionnaire may affect the willingness of some people to participate in the survey. As single questions are faster to answer than multi-item questionnaires those are often used for screening dental fear and in epidemiological studies. On the other hand, multi-item questionnaires give researchers more information and they are often used e.g., in clinical studies [52]. This health inquiry was answered on the internet, which might have made it easier for the students to participate, but some people may not want to answer questions concerning their health on the internet. In addition, an electronic questionnaire might result in a more valid estimation of dental fear than if it was asked, e.g., in connection with a clinical examination, in which those with high fear might not participate. In this study the question about tobacco use combined smoking and use of snuff. This question has not been validated; however, there was an almost identical prevalence of tobacco use in this study and in the Student Health Survey 2012 [41]. Because this study is cross-sectional, no causal interpretations can be made; and it is not possible to know e.g., whether dental fear increases need for dental treatment or vice versa.

## Conclusion

In conclusion, those with high dental fear seem to be at risk of having poor oral health habits, abnormal attitude to food and frequent need for treatment at dental check-ups. Individuals with high dental fear need special attention from dental teams as poor oral health habits and abnormal attitude to food may increase the risk that oral health will deteriorate. Dental teams should make efforts with fearful patients in helping them to find motivation for good oral health habits.
